# Kappa opioid receptors in the central amygdala modulate spinal nociceptive processing through an action on amygdala CRF neurons

**DOI:** 10.1186/s13041-020-00669-3

**Published:** 2020-09-18

**Authors:** Guangchen Ji, Volker Neugebauer

**Affiliations:** 1 Department of Pharmacology and Neuroscience, Texas Tech University Health Sciences Center, School of Medicine, 3601 4th St, Lubbock, TX 79430-6592 USA; 2 Center of Excellence for Translational Neuroscience and Therapeutics, Texas Tech University Health Sciences Center, Lubbock, TX USA; 3 Garrison Institute on Aging, Texas Tech University Health Sciences Center, Lubbock, TX USA

**Keywords:** Amygdala, Kappa opioid receptor, Spinal dorsal horn, Nociception, Optogenetics

## Abstract

The amygdala plays an important role in the emotional-affective aspects of behaviors and pain, but can also modulate sensory aspect of pain (“nociception”), likely through coupling to descending modulatory systems. Here we explored the functional coupling of the amygdala to spinal nociception. We found that pharmacological activation of neurons in the central nucleus of the amygdala (CeA) increased the activity of spinal dorsal horn neurons; and this effect was blocked by optogenetic silencing of corticotropin releasing factor (CRF) positive CeA neurons. A kappa opioid receptor (KOR) agonist (U-69,593) was administered into the CeA by microdialysis. KOR was targeted because of their role in averse-affective behaviors through actions in limbic brain regions. Extracellular single-unit recordings were made of CeA neurons or spinal dorsal horn neurons in anesthetized transgenic Crh-Cre rats. Neurons responded more strongly to noxious than innocuous stimuli. U-69,593 increased the responses of CeA and spinal neurons to innocuous and noxious mechanical stimulation of peripheral tissues. The facilitatory effect of the agonist was blocked by optical silencing of CRF-CeA neurons though light activation of halorhodopsin expressed in these neurons by viral-vector. The CRF system in the amygdala has been implicated in aversiveness and pain modulation. The results suggest that the amygdala can modulate spinal nociceptive processing in a positive direction through CRF-CeA neurons and that KOR activation in the amygdala (CeA) has pro-nociceptive effects.

## Introduction

The amygdala has emerged as an important node of the emotional-affective aspects of pain and pain modulation [1–6]. The central nucleus (CeA) serves major amygdala output functions and receives pain-related information through the spino-parabrachio-amygdala pathway as well as from thalamic and cortical regions through the basolateral amygdala network [2]. Importantly, synaptic plasticity in the CeA in different pain models has been linked to emotional responses such as vocalizations, aversive behaviors in conditioned place preference/aversion assays, and anxio-depressive behaviors [7–16]. The contribution of the amygdala to sensory aspects of pain such as hypersensitivity in pain models is less clear. Manipulations of amygdala activity provided evidence for dual pro- and anti-nociceptive effects [4, 17–26] but others had little, if any effect on hypersensitivity [15, 16, 27–32]. The current view is that distinct amygdala circuits and cell types serve different functions related to different aspects of pain.

Nociceptive plasticity in the spinal dorsal horn plays a critical role in pain-related hypersensitivity [33, 34]. However, there is little information about the modulation of dorsal horn activity by the amygdala, although amygdala neurons project to and can modulate descending pain control centers such as periaqueductal gray PAG [2, 35, 36]. A recent study showed that morphine injection into the CeA, but not anterior cingulate cortex (ACC), reduced the responses to spinal dorsal horn neurons to noxious mechanical stimulation in a neuropathic pain model [37]. And block of kappa opioid receptors (KOR) in the CeA restored the loss of diffuse noxious inhibitory control (DNIC) in a neuropathic pain model, implicating amygdala KOR in descending pain modulation.

Here we used a selective KOR agonist (U-69,593) to manipulate CeA neuronal activity and measure the consequences on spinal dorsal horn neurons. Interactions between the KOR and CRF systems have been linked to aversiveness, anxiety and stress responses [38], and amygdala CRF-CeA neurons project to brain areas involved in pain modulation such as PAG [39]. Therefore, we tested the contribution of CRF-CeA neurons to the spinal effects of KOR activation in the amygdala, using optogenetic silencing of CRF-CeA neurons.

## Materials and methods

### Animals

Male, hemizygous transgenic and wildtype Crh-Cre rats on Wistar background [39–41] (initial breeding pairs kindly provided by Dr. Robert Messing, UT Austin), 250–350 g at time of testing, were housed on a 12-h light-dark cycle with unrestricted access to food and water. On the day of the experiment, animals were acclimated to the laboratory for at least 1 h. All procedures were approved by the Institutional Animal Care and Use Committee (IACUC) at the Texas Tech University Health Sciences Center (TTUHSC) and conformed to the policies and recommendations of the National Institutes of Health (NIH) Guide for the Care and Use of Laboratory Animals.

### Experimental protocol

Single-unit recordings of CeA neurons were made in anesthetized naïve rats before, during and after administration of a KOR agonist (U-69,593) into the CeA by microdialysis (15 min). In some experiments, an AAV vector expressing halorhodopsin was injected into the CeA 4–5 weeks before the electrophysiology recordings to determine the effect of optical silencing of CRF-CeA neurons on the activity of CRF-CeA neurons. In these experiments, a recording electrode and a microdialysis or optical fiber were inserted into the CeA region. In another set of experiments, single-unit recordings of spinal dorsal horn neurons were made in anesthetized naïve rats before and during administration of U-69,593 into the CeA by microdialysis (15 min) and during optical silencing of CRF-CeA neurons while U-69,593 administration continued for another 15 min. For the optogenetic experiments, an AAV vector expressing halorhodopsin was injected into the CeA 4–5 weeks before. In these experiments, a microdialysis fiber and an optical fiber were inserted into the CeA.

### Systems electrophysiology

#### Amygdala

Extracellular single-unit recordings were made from neurons in the lateral-capsular division of the CeA in the right hemisphere as described previously [14, 32, 42, 43]. Rats were anesthetized with isoflurane (3–4% induction, 2% maintenance; precision vaporizer, Harvard Apparatus, Holliston, MA). Core body temperature was maintained at 37 °C with a homeothermic blanket system. Using a stereotaxic frame (David Kopf Instruments, Tujunga, CA), a craniotomy was performed at the sutura frontoparietalis level for the insertion of the recording electrode (glass-insulated carbon filament electrode, 4–6 MΩ) and a microdialysis probe for drug or vehicle administration (see “Intra-amygdala drug application by microdialysis”), or an optical fiber for delivering yellow (590 nm) light pulses (see “Optogenetics”). A recording electrode was inserted stereotaxically into the CeA with a microdrive (David Kopf Instruments) using the following coordinates: 2.3–2.8 mm caudal to bregma, 3.8–4.2 mm lateral to midline, 7–8 mm deep. The recorded signals (action potentials/spikes) were amplified, band-pass filtered (300 Hz to 3 kHz), and processed by an interface (1401 Plus; Cambridge Electronics Design, CED, Cambridge, UK). Spike2 software (version 4; CED) was used for spike sorting and data analysis. Spike size and configuration were monitored continuously. For each neuron, a spike template was created during a 5 min baseline recording period, capturing the waveform within set limits of variability for amplitude, duration, and rise time. Only those neurons were included in the study that showed a spike configuration that matched the preset template and could be clearly discriminated from activity in the background throughout the experiment. Neurons were identified by monitoring background activity and responses to search stimuli, i.e., compression of the contralateral hind paw at innocuous (100 g/6 mm^2^) and noxious (500 g/6 mm^2^) intensities with a calibrated forceps. Neurons were selected that had a receptive field in the hindpaw and were activated more strongly by noxious than innocuous mechanical stimuli. Neuronal activity was measured as spikes/s for 10 min (background activity in the absence of intentional stimulation) and then during mechanical test stimulation (compression of the paw for 15 s). The interval between the innocuous stimulus and the noxious stimulus was 15 s, and measurements were repeated about every 5 min. For net evoked responses, background activity (spikes/15 s) preceding the stimulus was subtracted from the total number of spikes during stimulation (15 s).

#### Spinal cord

Extracellular single-unit recordings were made from wide dynamic range (WDR) neurons in the left spinal dorsal horn as described previously [44, 45]. WDR neurons respond more strongly to stimuli of noxious than innocuous intensities. Rats were anesthetized with isoflurane (3–4% induction, 2% maintenance). Body temperature was maintained at 37 °C with a homeothermic blanket system. A small laminectomy at vertebral levels T13-L2 exposed the lumbar spinal segments. Then the animal was mounted in a stereotaxic frame (David Kopf Instruments), the dura mater was opened, and a small pool was formed with agar to cover the exposed spinal cord with mineral oil. A glass insulated carbon filament electrode (4–6 MΩ) was inserted perpendicularly to the spinal cord surface using a microdrive (David Kopf Instruments) to record the activity of WDR neurons in the deep dorsal horn (300–1000 μm) of the lumbar enlargement of the spinal cord (L5/6). Signals (background activity and evoked responses) were recorded and analyzed as described for the amygdala.

### Intra-amygdala drug application by microdialysis

A KOR agonist (U-69,593, Tocris Bioscience, R&D Systems, Minneapolis, MN) was administered stereotaxically into the CeA by microdialysis while neurons in the CeA or spinal dorsal horn were recorded. At least 1 h before recordings started, a microdialysis probe (CMA/Microdialysis 11, 240 μm diameter, 6 kDa; Solna, Sweden) was inserted stereotaxically into the CeA (1.8–2.3 mm caudal to bregma, 4.0–4.5 mm lateral to midline, 8.0 mm deep) at a 5^0^ angle to allow simultaneous positioning of the recording electrode or optical fiber (see Optogenetics). The probe was connected to an infusion pump (Harvard Apparatus) with polyethylene tubing. Artificial cerebrospinal fluid (ACSF; in mM: 117 NaCl, 4.7 KCl, 1.2 NaH2PO4, 2.5 CaCl2, 1.2 MgCl2, 25 NaHCO3, and 11 glucose) was continuously perfused through the fiber at 5 μl/min. ACSF served as control before and after administration of U-69,593. U-69,953 stock solution was diluted in ACSF to the final concentration (100 μM), which is 100-fold higher than the intended target concentration in the tissue due to the concentration gradient across the dialysis membrane and diffusion in the brain tissue [15, 45–48]. U-69,953 was administered into the CeA at a rate of 5 μl/min for at least 15 min to establish equilibrium in the tissue.

### Optogenetics

For optical silencing of CRF-CeA neurons, a viral vector (rAAV5/EF1a-DIO-eNpHR3.0-eYFP, 1 μl, 1012 units/100 μl) packaged by the vector core facility at the University of North Carolina, Chapel Hill, NC was injected into the CeA using a 5 μl Hamilton syringe (33 gauge) to express halorhodopsin in CRF neurons of the transgenic rats. Coordinates were as follows: 1.8–2.3 mm caudal to bregma, 4.0–4.5 mm lateral to midline, and 7.5 mm deep. 4–5 weeks were allowed for vector expression before the electrophysiology experiments. Light sensitive eNpHR3.0 (halorhodopsin) channels were activated with yellow (590 nm) laser light pulses (20 Hz, 1–5 mW, 3–15 min; Opto Engine LLC, Midvale, UT) through an optical fiber (200 μm diameter) inserted into the CeA. Pulsed optical activation of halorhodopsin in CRF-CeA neurons has been shown to silence these neurons [40].

### Histological verification of recording, drug administration and optical stimulation sites

**Fig. 1:**
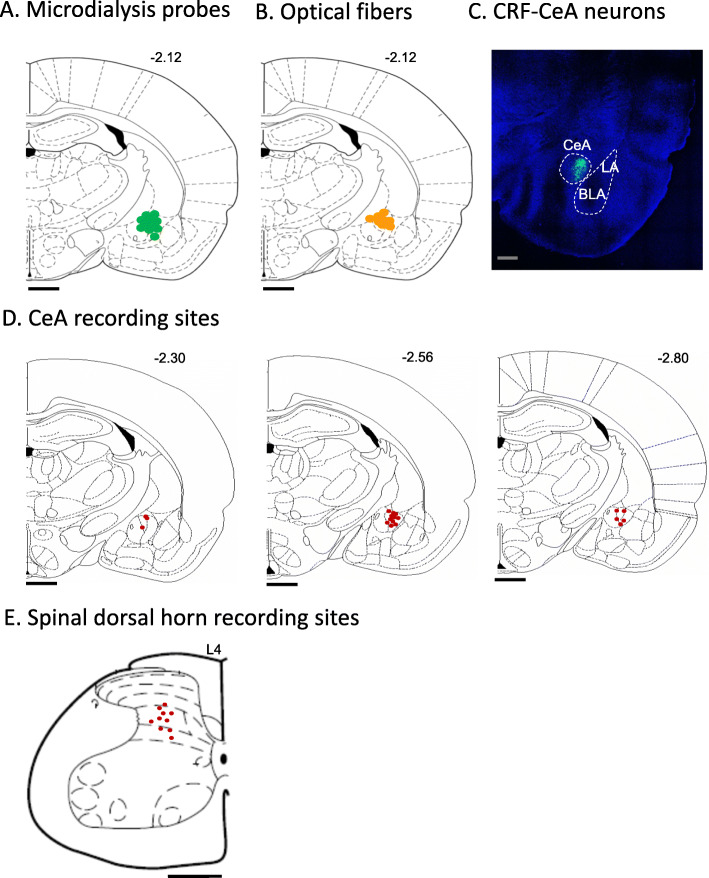
Recording, drug administration and optical stimulation sites. **a-d** Coronal brain slices. Numbers next to diagrams indicate distance from bregma. **a** Location of tips of microdialysis probes in the CeA for drug application (*n* = 12 sites). **b** Location of tips of optical fibers for optogenetic stimulation of CRF-CeA neurons (*n* = 8 sites). **c** Confocal image of eYFP fluorescence in CRF neurons in the CeA following viral vector (rAAV5/EF1a-DIO-eNpHR3.0-eYFP) injection to express halorhodopsin (see Materials and Methods, Optogenetics). **d** Site of electrolytic lesions indicating recording sites in CeA. **e** Spinal cord slice showing recoding sites in the dorsal horn of lumbar segment L4. **a-e** Scale bars, 500 μm

At the end of each experiment, the recording site in the CeA or spinal dorsal horn was marked with an electrolytic lesion by injecting DC (250 μA for 3 min) through the recording electrode. The brain or spinal lumbar enlargement was removed and submerged in 10% formalin and potassium ferrocyanide. Tissues were stored in 30% sucrose before they were frozen-sectioned at 50 μm and stained with hematoxylin and eosin. Lesion/recording sites and locations of the tips of the microdialysis and optical fibers were identified under bright-field microscopy and plotted on standard diagrams (Fig. 1).

### Statistical analysis

All averaged values are given as the mean ± SE. Statistical significance was accepted at the level *P* < 0.05. GraphPad Prism 7.0 software was used for all statistical analyses. Statistical analysis was performed on the raw data. Paired t-tests were used where appropriate. For multiple comparisons, ANOVA was used with Bonferroni posthoc tests.

## Results

Experiments were designed to test the hypothesis that KOR activation in the amygdala under normal conditions increases activity of spinal dorsal horn neurons through the activation of CRF neurons in the amygdala (CeA).

### Facilitatory effects of KOR activation on CeA neurons

**Fig. 2:**
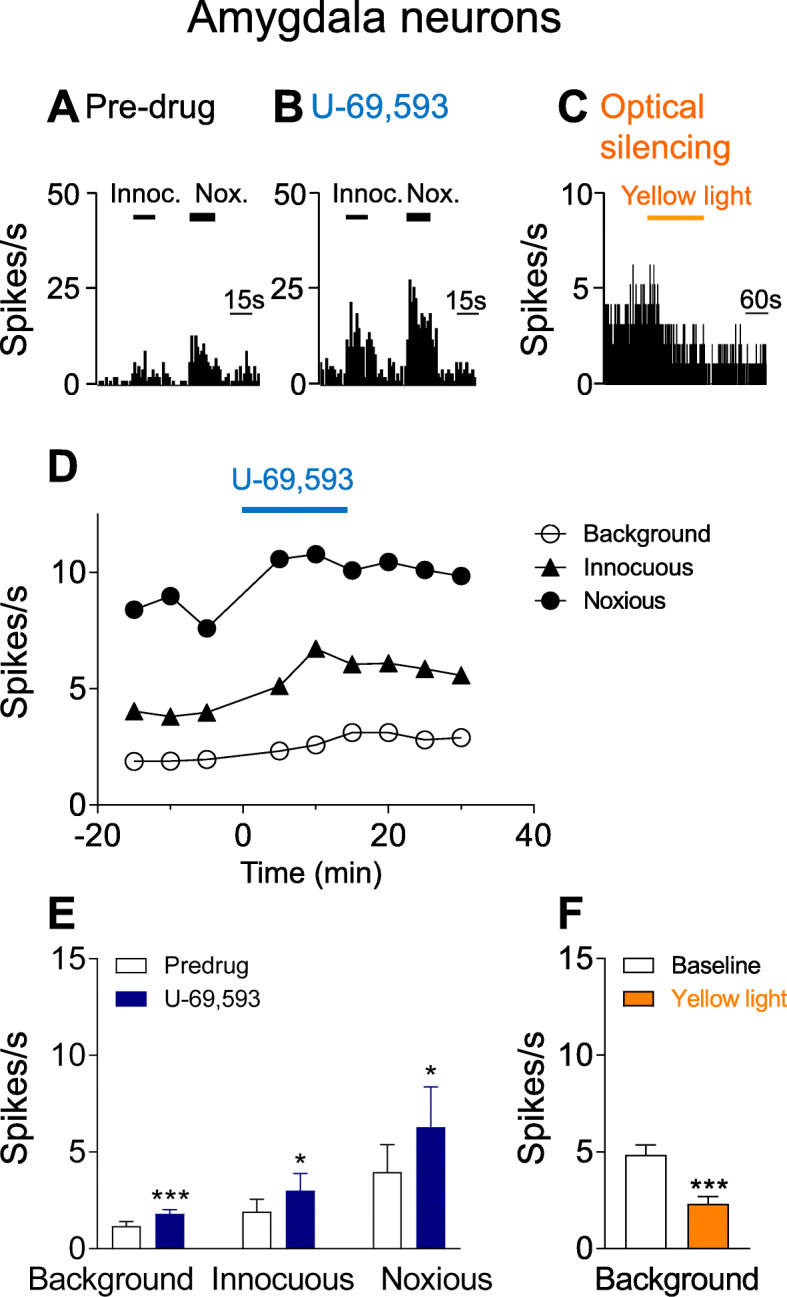
Facilitatory effects of KOR activation and inhibitory effects of CRF-CeA silencing on CeA neurons. Extracellular single-unit recordings of 19 neurons in the lateral and capsular CeA in anesthetized rats. **a-b** Peristimulus time histograms show action potentials (spikes) per second in an individual CeA neuron before (pre-drug control in ACSF) and during administration of U-69,593 (100 μM in microdialysis probe, 15 min) into the CeA. Background activity and evoked responses (see Materials Methods) increased. **c** Peristimulus time histogram shows effects of optical silencing of halorhodopsin expressing CRF-CeA neurons by yellow light pulses (590 nm, 20 Hz, 1–5 mW, 3 min) on action potential firing (see Materials and Methods, Optogenetics). **d** Time course of effects of U-69,593 in the same CeA neuron. **e** Summary of effects of U-69,593 in CeA neurons (*n* = 8).* *P* < 0.05, *** *P* < 0.001, compared to pre-drug ACSF, paired t-test. **f** Summary of effects of optical silencing of CRF-CeA neurons (*n* = 11). *** *P* < 0.001, compared to baseline before optical stimulation, paired t-test. **e, f** Bar histograms show mean ± SE for the sample of neurons

Extracellular single-unit recordings of 19 CeA neurons were made in 9 animals (1–2 neurons were studied in each animal). Recording sites in the lateral and capsular CeA are shown in Fig. 1d. Neurons were selected that responded more strongly to noxious than innocuous stimuli as in our previous studies [14, 49, 50]. In 8 CeA neurons the effect of a KOR agonist (U-69,593, 100 μM in microdialysis probe, 15 min) administered into the CeA by microdialysis was tested (Fig. 2). U-69,593 increased background activity and evoked responses to innocuous and noxious mechanical stimuli (compression of the hindpaw with a calibrated forceps; for details see Materials Methods, “Systems Electrophysiology”). Facilitatory effects were significant (*P* < 0.05 and *P* < 0.001, compared to pre-drug ACSF, paired t-test, *n* = 8; Fig. 2a, b, d, e). Drug application sites in the CeA are shown in Fig. 1a.

In another 11 CeA neurons the effect of optical silencing of CRF neurons was tested 4–5 weeks after injection of viral vector (rAAV5/EF1a-DIO-eNpHR3.0-eYFP) into CeA (see Fig. 1b; for details see Materials and Methods, “Optogenetics”). Optical silencing with yellow light pulses (590 nm, 20 Hz, 1–5 mW, 3–5 min) significantly decreased the background activity of CeA neurons (*P* < 0.001 compared to baseline, paired t-test, *n* = 11; Fig. 2c and f). Labelled CRF neurons in the CeA following AAV injection are shown in Fig. 1c. The data suggest that KOR activation has facilitatory effects on CeA neurons whereas silencing of CRF neurons has inhibitory effects.

### Facilitatory effects of KOR activation in CeA on spinal dorsal horn neurons were blocked by silencing CRF neurons in the CeA

**Fig. 3:**
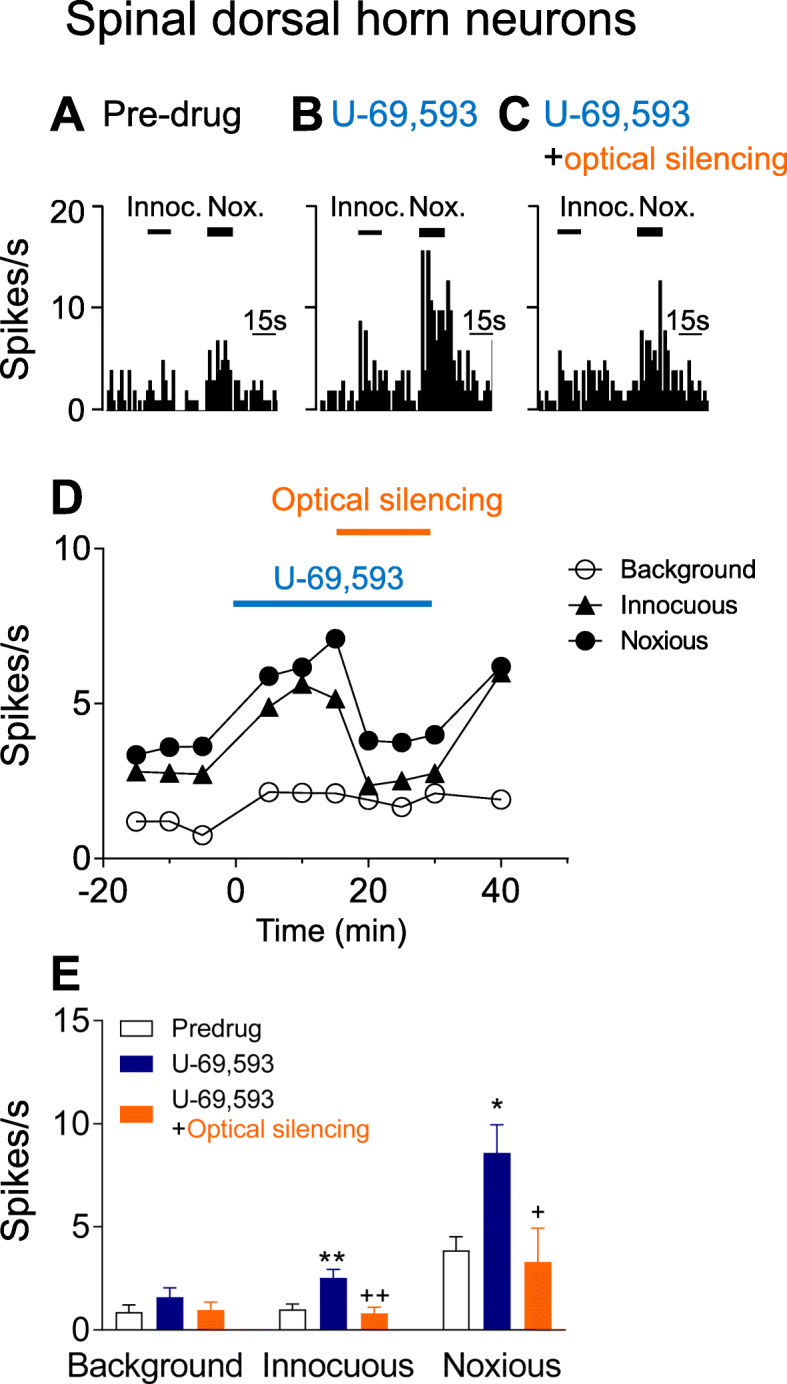
Facilitatory effects of KOR activation in CeA on spinal dorsal horn neurons were blocked by silencing CRF neurons in the Ce**A.** Extracellular single-unit recordings of 10 spinal dorsal horn WDR neurons in anesthetized rats. **a-c** Peristimulus time histograms show action potentials (spikes) per second in an individual spinal WDR neuron before (pre-drug control in ACSF; **A**) and during administration of U-69,593 (100 μM in microdialysis probe, 15 min; **B**) into the CeA and during continued administration of U-69,593 while CRF-CeA neurons were silenced optogenetically (590 nm, 20 Hz, 1–5 mW, 3 min; **C**). Optical silencing of CRF-CeA neurons inhibited the facilitatory effects of U-69,593. **d** Time course of effects of U-69,593 in the CeA and optical silencing of CRF-CeA neurons on the same WDR neuron. **e** Summary of effects of U-69,593 in the CeA and optical silencing of CRF-CeA neurons on WDR neurons (*n* = 10). * *P* < 0.05, ** *P* < 0.01, compared to pre-drug ACSF; ^+^ *P* < 0.05, ^++^ *P* < 0.01, compared to U-69,593 alone; ANOVA with Bonferroni posthoc tests. See text for results of ANOVA. Bar histograms show mean ± SE for the sample of neurons

Using this information, we studied the effects of amygdala KOR activation on spinal dorsal horn neurons and examined the contribution of CRF neurons in the CeA. Extracellular single-unit recordings of 10 spinal dorsal horn WDR neurons were made in 7 animals (Fig. 3). Recording sites in the deep dorsal horn of the left side of the lumbar cord are shown in Fig. 1e. WDR neurons responded more strongly to noxious than to innocuous stimuli applied to the hind paw (see Materials and Methods, “Systems Electrophysiology”). Administration of U-69,593 (100 μM in microdialysis probe, 15 min) into the right CeA by microdialysis significantly increased responses to innocuous (F_2,23_ = 7.43, *P* < 0.01) and noxious stimuli (F_2,23_ = 6.103, *P* < 0.05, compared to pre-drug ACSF, ANOVA with Bonferroni posthoc tests, *n* = 10; Fig. 3a, b, d, e). In 6 of these neurons, the effect of optical silencing (590 nm, 20 Hz, 1–5 mW, 15 min) of CRF-CeA neurons was tested during continued administration of U-69,593 (100 μM in microdialysis probe) into the CeA for another 15 min (Fig. 3c, d, e). Optical silencing of CRF-CeA neurons significantly inhibited the facilitatory effects of U-69,593 on responses to innocuous (*P* < 0.01, F_2,23_ = 7.43) and noxious stimuli (*P* < 0.05, F_2,23_ = 6.103; compared to U-69,593 alone; ANOVA with Bonferroni posthoc tests, *n* = 6). Pulsed activation of halorhodopsin was used (see Methods, Optogenetics) that was shown before to silence CRF-CeA neurons [40]. Effects of optical silencing were reversible. Effects of U-69,593 and optical silencing on background activity did not reach the level of statistical significance (F_2,23_ = 0.9726, ANOVA).

## Discussion

The data presented here show that pharmacological activation of KOR in the CeA with U-69,593 increases activity of neurons in the CeA and in the spinal dorsal horn, suggesting a functional connection and positive correlation between neuronal activity in these regions. This connectivity was disrupted when CRF neurons in the CeA were silenced optogenetically, which suggests an important contribution of CRF neurons to amygdala output coupled to descending modulation of spinal nociceptive processing.

We focused on KOR to explore the amygdala-spinal cord connection because KOR in the amygdala have been linked to aversiveness, anxiety and stress responses [38, 51–54] as well as to averse-affective behaviors in stress- or injury-induced pain conditions [16, 55–57]. KOR is expressed in the CeA at particularly high levels [58]. We used a selective KOR agonist (U-69,593) [59] at a concentration that was based on data in the literature from electrophysiological studies in brain slices (see [60, 61]. U-69,593 has been shown to decrease synaptic inhibition of medial CeA neurons through a presynaptic action [60, 61]. U-69,593 also produced an outward current in medial CeA neurons, indicative of a postsynaptic inhibitory effect [62, 63]. Increased activity of lateral-capsular CeA neurons by U-69,593 in the present study is consistent with disinhibition observed in the brain slice studies [60, 61]. The facilitatory effects of KOR activation in the CeA on spinal dorsal horn responses suggest a positive correlation and functional connection between amygdala activity and spinal nociceptive processing.

We studied KOR function in the right CeA because of evidence for right-hemispheric lateralization of CeA function [49, 64, 65] and KOR function in the CeA related to pain modulation [16, 56, 57, 66]. For example, blockade of KOR in the right, but not left, CeA restored diffuse noxious inhibitory control (DNIC), a measure of descending pain control [56, 57].

Evidence for functional links between amygdala KOR and CRF systems [38] let us to explore the contribution of CRF neurons to the descending modulation of nociceptive processing. CRF neurons in the CeA project to extra-amygdalar targets to promote averse-affective behaviors [39, 41, 67–69]. Amygdala CRF functions are under tonic inhibitory control of KOR as shown with a KOR antagonist that enhanced the synaptic effects of CRF on medial CeA neurons [70]. We used optogenetics to silence CRF neurons in transgenic Crh-Cre rats and found inhibitory effects on CeA neurons as well as inhibition of the facilitatory effects of U-69,593 on spinal neurons. The CeA cell type recorded in this in vivo study is not known but it is possible that they were CRF neurons that were inhibited directly with optical silencing. This scenario is supported by the fact that CRF-CeA neurons project to brainstem areas involved in descending pain modulation such as the PAG [39]. Silencing of CRF neurons could also have effects on other types of CeA neurons that form long range projections including those containing somatostatin [71]. CRF neurons can excite [72] whereas somatostatin neurons inhibit [71] PAG neurons. In addition to coupling to brainstem centers involved in descending pain modulation, the amygdala CeA could influence spinal nociceptive processing through indirect influences on cortical regions such as ACC [73, 74] via cholinergic neurons in substantia innominata and nucleus basalis of Meynert [75]. CeA neurons do not directly project to cortical regions; main amygdala input to ACC arises from the basolateral amygdala [76]. Therefore, the amygdala can exert facilitatory influences on spinal nociceptive processing either through ascending or descending output to pain control systems [73, 77, 78] by activating facilitatory or inhibiting inhibitory modulation. This remains to be determined.

On a technical note, prolonged application of U-69,593 does not produce desensitization of CeA neurons [60]. This is important because our experimental protocol tested optical silencing of CRF-CeA neurons on the facilitatory effect of prolonged administration of U-69,593 on spinal nociceptive processing. Therefore, reversal of the effects of U-69,593 by optical silencing of CRF-CeA neurons was not due to desensitization. Finally, pulsed optical activation of halorhodopsin has been reported to evoke rebound spiking after hyperpolarization in certain cell types and brain regions [79, 80], which may not result in the desired silencing of neuronal activity. However, pulsed optical activation of halorhodopsin silenced CRF-CeA neurons without rebound spiking [40], which could be due to cell type, region and possibly species specific differences.

## Conclusion

This short communication provides important novel information about amygdalo-spinal interactions and the contribution of amygdala KOR and CRF systems. The data support the concept of a positive correlation and functional link between amygdala (CeA) and spinal nociceptive processing. Details of the neural circuitry and cell-types remain to be determined.

## Data Availability

All data generated or analyzed during this study are included in this published article. Data files. used for this manuscript are available via a direct and reasonable request to the corresponding. author and approval from Texas Tech University Health Sciences Center (TTUHSC).

## Abbreviations

ACC: Anterior cingulate cortex
BLA: Basolateral nucleus of the amygdala
CeA: Central nucleus of the amygdala
CRF: Corticotropin releasing factor
KOR: Kappa opioid receptor
PAG: Periaqueductal grey
